# Leptomeningeal Carcinomatosis: A Case Report and Literature Review

**DOI:** 10.7759/cureus.26790

**Published:** 2022-07-12

**Authors:** Mason Hinke, Alexandra Skovran, Nathaniel Dusini, Sofiya Azim

**Affiliations:** 1 Internal Medicine, Hennepin Healthcare, Minneapolis, USA; 2 Internal Medicine, Freeman Health, Joplin, USA

**Keywords:** leptomeningeal carcinomatosis (lmc) cerebrospinal fluid (csf), intramedullary spinal cord metastasis, central nervous system metastasis, colon cancer, lung cancer

## Abstract

Leptomeningeal carcinomatosis (LC) is a rare complication of malignant tumors that involves metastasis to the meninges surrounding the brain and/or spinal cord. The incidence of LC appears to be increasing, which has been attributed to increased survival times of cancer patients and increased diagnostic sensitivity. In this case, we discuss a patient with a history of colon cancer and lung cancer who was admitted with multiple cranial nerve palsies and sensory deficits. An MRI with contrast showed multiple enhancing intracranial lesions with leptomeningeal enhancement. Neurology and neurosurgery were consulted, and the patient was ultimately discharged to hospice.

## Introduction

Leptomeningeal carcinomatosis (LC) is a rare complication of malignant tumors that involves metastasis to the meninges surrounding the brain and/or spinal cord. LC is seen in up to 8% of solid malignancies and 15% of hematologic cancers [1]. The incidence of LC appears to be increasing, which has been attributed to increased survival times of cancer patients and increased diagnostic sensitivity [1]. Many solid tumors have been implicated in the development of LC, the most common being breast, lung (primarily small cell), and melanoma [1]. Patients with LC typically develop symptoms over days to weeks, often presenting with non-specific symptoms such as headache and nausea [2]. All patients suspected of having LC should undergo a thorough neurologic exam and contrast-enhanced magnetic resonance imaging (MRI), if possible. A lumbar puncture (LP) is often recommended but is not necessary if there are definitive findings on imaging. Patients that do undergo an LP should have the cerebrospinal fluid (CSF) sent for cell count, differential, total protein, glucose, and cytology [3]. False-negative CSF cytology can be seen in up to 36% of cases [3]. Clinical suspicion must remain high in patients with neurologic symptoms and a history of cancer.

## Case presentation

We discuss the case of a 74-year-old male with a history of colon cancer, T3N1b, stage IIIB, who underwent a right hemicolectomy in 2011. Pathology from the procedure was consistent with invasive adenocarcinoma with two of 48 lymph nodes involved. He received adjuvant capecitabine (Xeloda) and was monitored with routine surveillance. As part of the scheduled imaging, a 5 mm nodule was noted in the right upper lobe on chest CT in 2016. The case was discussed with interventional radiology and cardiothoracic surgery and surgery was deferred, as the lesion was initially determined to be too small. The nodule subsequently increased to 12 mm over a one-year span and the patient underwent a right upper lung biopsy that showed benign lung tissue with focal non-caseating granulomatous inflammation.

A repeat chest CT in 2018 showed interval growth of the nodule. A PET scan was ordered and revealed increased fluorodeoxyglucose (FDG) uptake, and a repeat biopsy was performed and was consistent with squamous cell carcinoma. The patient elected against surgery and underwent stereotactic body radiation therapy (SBRT) with partial response. In 2019, two additional densities were noted in the left lower lung and left middle lung and this was also treated with SBRT. Routine surveillance images through 12/2021 showed a favorable response to radiation and no progression of the densities was observed.

In March 2022, the patient presented to the hospital with a one-week history of right lower extremity weakness, left-sided facial droop, and loss of bladder control. He was seen at an outside facility and originally diagnosed with Bell’s palsy. He then came to the emergency department due to ongoing weakness and an inability to care for himself. Labs on admission were notable for sodium of 129 mEq/L and a procalcitonin of 0.05 ng/mL. Urinalysis showed 1+ ketones and positive leukocyte esterase.

On a physical exam, he was noted to have right facial numbness and left-sided facial droop. He was unable to completely close his left eye. Strength was 4/5 in the left upper extremity, 5/5 in the right upper extremity, 2/5 in the right lower extremity, and 4/5 in the left lower extremity. He was also noted to have decreased sensation below the level of T8.

**Figure 1:**
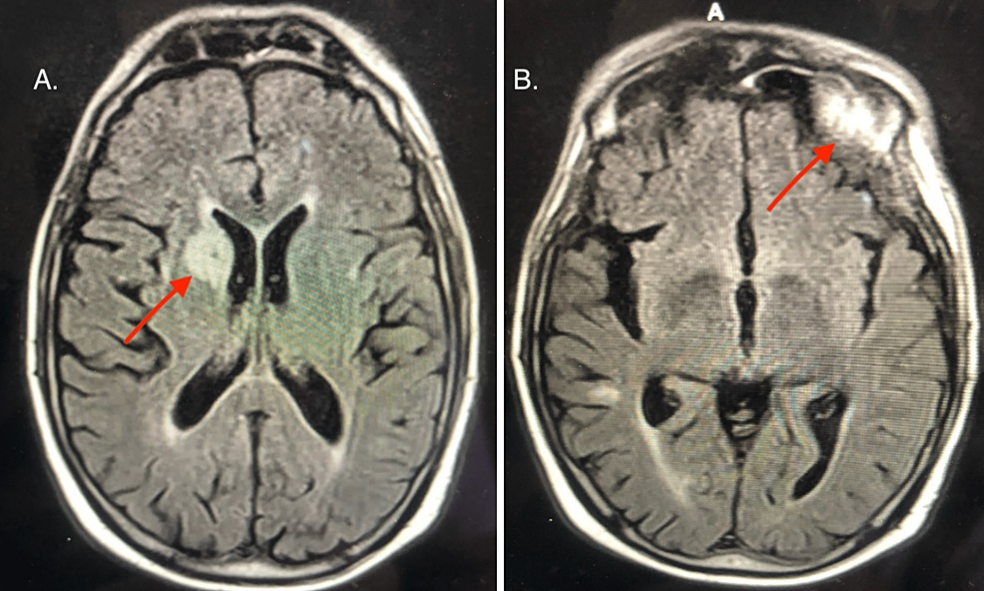
MRI with contrast revealed multiple enhancing intracranial lesions with leptomeningeal enhancement at the posterior fossa consistent with CNS metastasis 1A shows a paraventricular mass while 1B shows a mass in the left frontal lobe.

**Figure 2:**
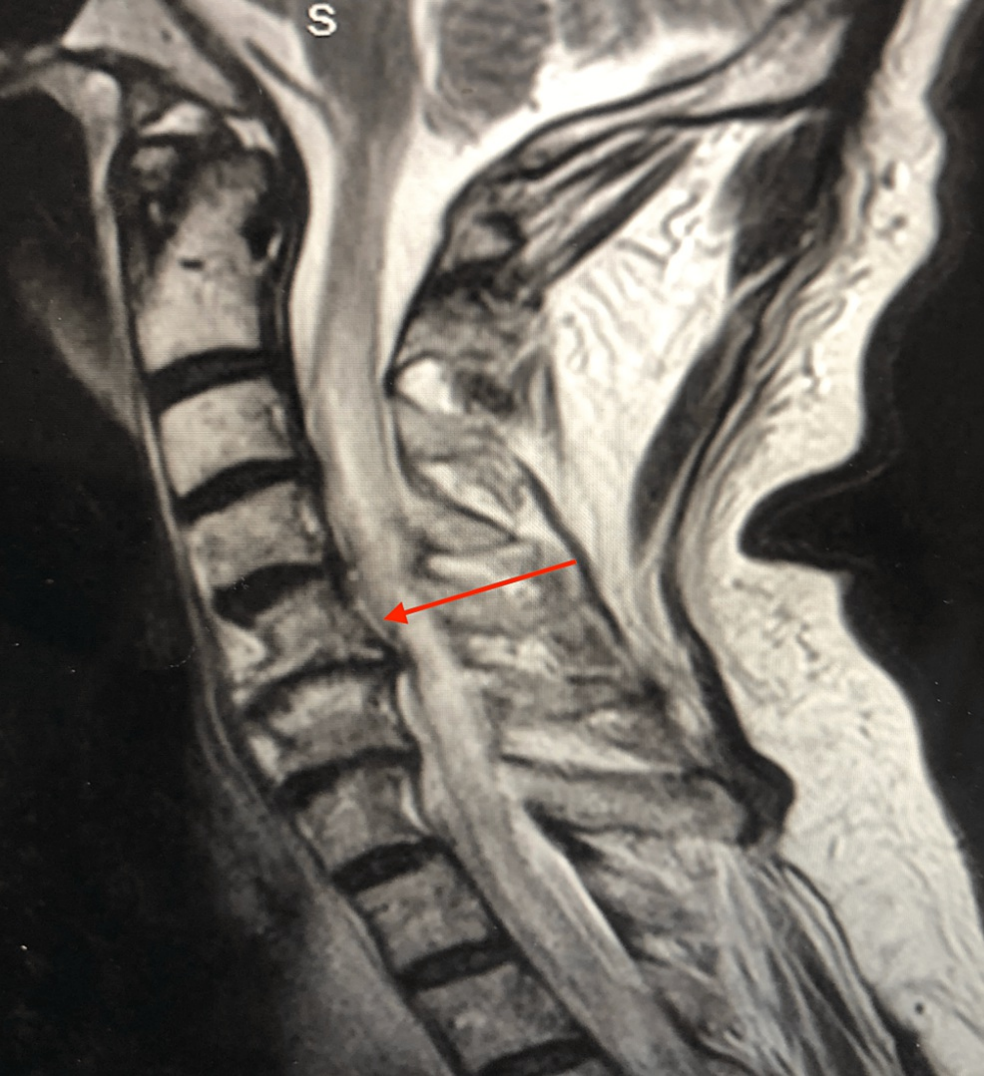
MRI with contrast of the cervical spine showing intraspinal metastasis

Given the concern for stroke vs multiple cranial nerve palsy, neurology was consulted. Brain MRI without contrast was ordered, which showed no evidence of acute infarction but a probable area of old lacunar infarction. An MRI with contrast showed multiple enhancing intracranial lesions with leptomeningeal enhancement at the posterior fossa consistent with CNS metastasis (Figure 1). Imaging of the cervical, thoracic and lumbar spine was ordered, which showed intramedullary and intradural extramedullary metastasis of the cervical and upper thoracic spine and leptomeningeal metastasis in the lumbar spine (Figure 2). Lumbar puncture was deferred due to the patient being on clopidogrel.

The patient was started on dexamethasone 4 mg every six hours with minimal improvement in symptoms. The case was discussed with neurosurgery, who deemed the patient would not benefit from any surgical intervention. The case was also discussed with hematology-oncology and given the limited functional status of the patient, he was deemed to not be a good candidate for intrathecal chemotherapy. It was inferred that the origin of LC was likely from his squamous cell lung cancer, despite the fact that he was previously treated with SBRT [4].

After discussion with the family and palliative care, the patient was transitioned to hospice. He was discharged home with dexamethasone 4 mg twice daily and morphine 4 mg every two hours as needed. As of two months post-discharge, the patient remained in hospice care.

## Discussion

Leptomeningeal carcinomatosis is a rare complication of late-stage malignancy that is becoming increasingly recognized. While breast cancer, lung cancer, and melanoma most commonly metastasize to the meninges, other solid and hematologic cancers have the potential to cause this devastating disease [5]. Initial presentation can be non-specific, often with patients presenting with nausea, vomiting, and headache. Clinical suspicion should be high in all patients with known metastatic cancer who present with new neurologic symptoms. A thorough neurologic exam should be performed and all patients suspected of LC should undergo an MRI of the brain and full spinal cord [2]. Leptomeningeal enhancement is the characteristic finding on MRI and visualization is generally enough to establish a diagnosis without cytology [5]. MRI has a sensitivity ranging from 76%-100% in solid tumor malignancy but decreases with hematologic tumors [2]. The gold standard for diagnosis of LC is CSF, demonstrating the presence of malignant cells. However, positive cytology is only seen in 50%-60% of cases [6]. The low sensitivity is attributed to low volumes, delays in processing, and examiner experience [6]. Serial CSF sampling has been suggested to improve the sensitivity, as some studies have found an initial false-negative cytology rate of up to 50%, however, this predisposes patients to a new set of complications [5]. Therefore, MRI imaging suggestive of LC, together with the clinical picture, is often all that is required for diagnosis.

The prognosis of leptomeningeal carcinomatosis remains poor, with most patients expiring within a few months of diagnosis. The median survival time varies slightly based on underlying malignancy. Breast cancer is estimated to be 3.5-4.4 months, lung cancer is between three and six months, and melanoma has the worst prognosis at 1.7-2.5 months. Treatment is aimed at prolonging survival and maintaining the quality of life by delaying neurologic deterioration. The symptoms at onset are often irreversible and rarely improve with treatment [7]. Anticonvulsants can be used as needed and selective-serotonin reuptake inhibitors (SSRI) or stimulants can be used in patients with significant depression and fatigue [3].

Current therapies involve intrathecal pharmacotherapy, systemic pharmacotherapy, and radiotherapy. Intrathecal therapy is widely used, however, studies have shown that there is limited penetration into solid tumor lesions, and it may cause CSF outflow obstructions as well as aseptic meningitis. Corticosteroids can aid in the treatment of aseptic meningitis, and radiotherapy has been used to decrease outflow obstructions and increase the circulation of the intrathecal agents [2]. The three drugs currently used for intrathecal treatment are methotrexate, liposomal cytarabine, and thiotepa. Current studies are underway evaluating the use of intrathecal trastuzumab in patients with LC from Her2-positive breast cancer metastasis [7]. Current treatment guidelines from the European Association of Neuro-Oncology (EANO) - European Society for Medical Oncology (ESMO) recommend targeting therapy based on pathologic and molecular tumor markers, general health status, neurologic status, and available therapeutic options for extra central nervous system disease [8].

## Conclusions

In this case report, we discussed the presentation of a 74-year-old male with a history of lung cancer and colon cancer who was admitted with neurological deficits and ultimately diagnosed with LC. The prognosis of patients with LC remains poor and treatment is primarily focused on prolonging quality of life. There are numerous ongoing studies looking at new chemotherapy regimens to prolong survival, however, it is important to consider the use of anticonvulsants, analgesics, and anxiolytics, when needed, to provide optimal and holistic care.
